# Mitochondrial Protection by Trifolirhizin Alleviates Primary Sjögren’s Syndrome and Liver Injury via Coordinated Suppression of the ROS/cGAS-STING Pathway

**DOI:** 10.3390/antiox15070814

**Published:** 2026-06-28

**Authors:** Haotian Li, Man Han, Rouman Zhang, Congmin Xia, Jianqin Yang, Yanjun Liu, Yuping Zhao, Quan Jiang

**Affiliations:** 1 Institute of Basic Theory of Traditional Chinese Medicine, China Academy of Chinese Medical Sciences, Beijing 100700, Chinazhaoyp@ibtcm.ac.cn (Y.Z.); 2 Department of Digestive Disorders, Beijing Key Laboratory of Diagnosis and Treatment of Functional Gastrointestinal Diseases of Traditional Chinese Medicine, Beijing 100102, China; 3 Capital Institute of Rheumatology and Immunology of Integrated Medicine, Beijing 100032, China; 4 Precision Al in Rheumatology Network, Beijing 100032, China

**Keywords:** trifolirhizin, ROS, cGAS-STING, autoimmune liver injury, mitochondrial protection, primary Sjögren’s syndrome

## Abstract

Background: Autoimmune diseases such as primary Sjögren’s syndrome and type 1 diabetes are frequently complicated by hepatic injury, yet therapies that simultaneously target inflammation and parenchymal damage remain limited. Mitochondrial dysfunction with excessive reactive oxygen species (ROS) production drives a self-amplifying pathogenic loop by activating the cGAS-STING innate immune pathway. We previously observed that a Chinese herbal formula preserved mitochondrial ultrastructure in autoimmune NOD mice, and computational screening identified trifolirhizin—a natural pterocarpan flavonoid—as the candidate active constituent mediating this protection. Here, we investigated the hepatoprotective effects and underlying mechanisms of trifolirhizin in autoimmune-associated liver injury. Methods: Female NOD mice received trifolirhizin (5, 10, or 20 mg/kg/day) for four weeks, with C57BL/6J mice as healthy controls. Hepatic histopathology, inflammatory cytokines, mitochondrial ultrastructure (TEM), mitochondrial membrane potential (ΔΨm), and ROS levels were evaluated. Integrated transcriptomic and metabolomic profiling was performed to unbiasedly characterize protective mechanisms. In vitro, H_2_O_2_-induced oxidative stress was established in HepG2 cells. Cells were treated with trifolirhizin (15–25 µM) and assessed for antioxidant enzyme activities, ΔΨm, ROS production, glycolytic and mitochondrial respiration (Seahorse analysis), and cGAS-STING pathway protein expression. Pharmacological rescue experiments using the cGAS agonist cGAMP were conducted to test pathway dependency. Results: Trifolirhizin dose-dependently alleviated hepatic pathological damage and reduced pro-inflammatory cytokine levels in NOD mice. Multi-omics profiling revealed that oxidative stress responses, the mitochondrial electron transport chain, and glutathione metabolism were the most significantly restored pathways. Trifolirhizin preserved mitochondrial ultrastructure, restored ΔΨm, and attenuated ROS accumulation both in vivo and in vitro. Functionally, Seahorse analysis demonstrated that trifolirhizin rescued overall cellular bioenergetics, restoring both glycolytic capacity and mitochondrial respiratory parameters (basal respiration, ATP production, maximal respiration, and spare respiratory capacity). Mechanistically, trifolirhizin suppressed the cGAS-STING-TBK1-IRF3 axis, as evidenced by reduced expression of cGAS, p-STING, ZBP1, p-TBK1, and p-IRF3. Importantly, the cGAS agonist cGAMP abrogated the protective effects of trifolirhizin, confirming that the cGAS-STING pathway is functionally required for its action downstream of mitochondrial protection. Conclusion: Trifolirhizin attenuates liver injury in the nod mouse by preserving mitochondrial integrity, maintaining cellular energy metabolism, and thereby suppressing the ROS/cGAS-STING inflammatory cascade. These findings position trifolirhizin as a promising mitochondria-targeted therapeutic candidate for pSS-related hepatic complications and provide a mechanistic framework for discovering active compounds from mitochondrially active herbal formulations.

## 1. Introduction

Autoimmune diseases, such as primary Sjögren’s syndrome (pSS), are chronic systemic disorders driven by aberrant activation of the innate and adaptive immune system, often leading to multi-organ damage. Although pSS primarily targets exocrine glands, causing dry mouth (xerostomia) and dry eyes (keratoconjunctivitis sicca), systemic extra-glandular manifestations are common. Hepatic involvement is one of the most frequent organ complications, reported in 27–49% of pSS patients, and is characterized by inflammatory infiltration, hepatocyte damage, and progressive dysfunction, which significantly impairs patient prognosis and quality of life [1,2]. Current clinical management primarily relies on glucocorticoids and non-specific immunosuppressants, which are limited by long-term toxicity and lack of targeted efficacy. Despite its clinical relevance, the mechanisms driving pSS-associated liver injury remain poorly understood, and no targeted therapies are currently available. Investigating this specific extra-glandular complication is therefore of considerable translational significance.

Mitochondrial dysfunction and excessive reactive oxygen species (ROS) accumulation are now recognized as central pathogenic events linking immune dysregulation to tissue injury in autoimmune settings [3,4]. As organelles of high abundance in metabolically active tissues, mitochondria serve not only as the primary source of cellular ROS but also as early targets of oxidative damage. Disruption of mitochondrial membrane potential (ΔΨm) and electron transport chain integrity promotes the release of mitochondrial DNA (mtDNA) into the cytosol, where it acts as a potent damage-associated molecular pattern. Cytosolic mtDNA, together with elevated ROS, synergistically activates cyclic GMP-AMP synthase (cGAS), a critical cytosolic DNA sensor. This triggers the synthesis of cyclic GMP-AMP (cGAMP), which in turn activates stimulator of interferon genes (STING). Activated STING recruits and phosphorylates TANK-binding kinase 1 (TBK1), leading to phosphorylation of interferon regulatory factor 3 (IRF3) and the subsequent production of type I interferons and pro-inflammatory cytokines [5,6]. This self-amplifying loop of “ROS overproduction → mitochondrial damage → cGAS-STING hyperactivation” has been functionally implicated in several autoimmune and inflammatory liver diseases, positioning the ROS/cGAS-STING axis as a promising therapeutic target [7,8]. Given that hepatocytes are exceptionally rich in mitochondria and highly susceptible to oxidative stress, the liver represents an ideal model organ in which to investigate interventions aimed at preserving mitochondrial integrity and interrupting this pathogenic cascade.

Trifolirhizin is a natural pterocarpan flavonoid isolated from leguminous plants, with documented antioxidant, anti-inflammatory, and anti-tumor activities [9]. Our interest in this compound was initiated by an unexpected observation in our prior work: a Chinese herbal formula demonstrated remarkable preservation of mitochondrial ultrastructure in non-obese diabetic (NOD) mice, a spontaneous model recapitulating features of both pSS and T1DM. Following reviewer recommendations to identify the active constituent(s) responsible, we performed targeted computational screening of the flavonoid constituents within the formula, when molecular docking techniques have become increasingly important tools for target discovery [10].

Trifolirhizin emerged as a top candidate, displaying favorable molecular docking scores with key targets involved in mitochondrial quality control and redox regulation [11], findings further supported by molecular dynamics simulations. Despite these bioinformatic predictions and the known pharmacological profile of trifolirhizin, its role in autoimmune-associated organ injury—and specifically its effects on mitochondrial function—remains entirely unexplored. We hypothesized that trifolirhizin may exert protective effects in pSS and its associated hepatic complications by modulating mitochondrial integrity and the ROS/cGAS-STING signaling pathway.

In this study, we employed the NOD mouse, a well-characterized spontaneous model of primary Sjögren’s syndrome (pSS), to investigate the hepatoprotective effects of trifolirhizin in vivo. Given its high mitochondrial content and susceptibility to immune-mediated injury, the liver was selected as the target organ. Treatment was initiated at 8 weeks of age and continued for 4 weeks, a time window that precedes the onset of overt type 1 diabetes in this strain. Weekly blood glucose monitoring confirmed that all NOD groups remained normoglycemic throughout the experiment, thereby excluding metabolic improvement as a confounding factor. The pSS-like phenotype was verified by stimulated salivary flow measurements. We systematically evaluated hepatic histopathology, inflammatory cytokine profiles, mitochondrial ultrastructure, ΔΨm, and ROS levels. To gain unbiased and systems-level mechanistic insight, we performed integrated transcriptomic and metabolomic profiling of liver tissues from NOD mice. Parallel in vitro experiments using H_2_O_2_-induced oxidative stress in HepG2 cells were designed to dissect the underlying molecular machinery. Seahorse extracellular flux analysis was employed to quantitatively assess glycolytic function and mitochondrial respiratory capacity, and Western blotting was used to examine core components of the cGAS-STING-TBK1-IRF3 pathway. Pharmacological rescue experiments with the cGAS agonist cGAMP were conducted to validate the functional relevance of this signaling axis. Our findings identify trifolirhizin as a novel mitochondrial-protective agent that attenuates pSS-associated liver injury and provide mechanistic insights into the therapeutic targeting of the ROS/cGAS-STING pathway in autoimmune disease complications.

## 2. Materials and Methods

### 2.1. Experimental Animals

#### 2.1.1. Animal Grouping and Drug Administration

Female NOD/ShiLtJ mice (6 weeks old) and age-matched C57BL/6J controls were purchased from Beijing Viewsolid Life Technology Co., Ltd. (Beijing, China) and housed under specific pathogen-free conditions with standard chow and autoclaved water ad libitum. After one week of acclimatization, the Sjögren’s syndrome-like phenotype was verified by salivary flow measurement; mice with confirmed salivary hypofunction were enrolled and randomly allocated into 4 groups (n = 8 each) at 8 weeks of age. To monitor metabolic status without assuming all NOD mice become diabetic during this early window, fasting blood glucose was measured weekly; no significant inter-group differences were observed, and all mice remained normoglycemic throughout the 4-week treatment period. All mice received daily intragastric administration with a fixed gavage volume of 0.1 mL per mouse per day for 4 consecutive weeks. The final DMSO concentration was consistent across all groups to ensure Trifolirhizin dosage was the only variable. The grouping details were as follows: Blank Control Group: C57BL/6J mice, administered with 0.1 mL of vehicle (5% DMSO in sterile normal saline) daily; NOD Model Group: NOD mice, administered with 0.1 mL of the same 5% DMSO vehicle daily; Trifolirhizin Low-dose Group: NOD mice, administered with 0.1 mL of Trifolirhizin suspension daily, equivalent to 5 mg/kg per mouse per day; Trifolirhizin Medium-dose Group: NOD mice, administered with 0.1 mL of Trifolirhizin suspension daily, equivalent to 10 mg/kg per mouse per day; Trifolirhizin High-dose Group: NOD mice, administered with 0.1 mL of Trifolirhizin suspension daily, equivalent to 20 mg/kg per mouse per day. The doses of trifolirhizin used in this study were selected according to previously reported studies [12]. The trifolirhizin used in this study was obtained from Beijing Solarbio Science & Technology Co., Ltd. (CAT: IT5910, HPLC ≥ 98%, Beijing, China).

At the end of the experiment, mice were deeply anesthetized with isoflurane, and blood was collected via retro-orbital cardiac puncture. Subsequently, mice were euthanized by CO_2_ exposure, and the liver and other target tissues were harvested. All animal experimental protocols were approved by the Animal Ethics Review Committee of the Institute of Traditional Chinese Medicine Basic Theory, Chinese Academy of Chinese Medical Sciences; Approval Code: IBTCMCACMS21-2510-09.

#### 2.1.2. Preparation of Trifolirhizin Suspension

Trifolirhizin was accurately weighed and completely dissolved in sterile DMSO. The DMSO solution was then diluted with sterile normal saline to obtain final concentrations of 50 mg/mL, 100 mg/mL, and 200 mg/mL Trifolirhizin suspensions, with a final DMSO concentration of 5% (*v*/*v*) in all formulations. The vehicle for control and model groups was prepared with the same volume of DMSO diluted in sterile normal saline to ensure consistent DMSO exposure across all experimental groups.

#### 2.1.3. Transmission Electron Microscopy (TEM), Hematoxylin and Eosin Staining (HE), Enzyme-Linked Immunosorbent Assay (ELISA) and Western Blot (WB)

These experimental procedures are consistent with those in our previous work [13,14]. The following primary antibodies were used: cGAS (Proteintech, 29958-1-AP; 1:1000, Rosemont, IL, USA); STING (ABclonal, A21051; 1:5000, Woburn, MA, USA); p-STING (Affinity, AF7416; 1:1000, San Francisco, CA, USA); ZBP1 (Servicebio, GB113835; 1:500, Wuhan, China); IRF3 (CST, 4302; 1:1000, Chicago, TX, USA); p-IRF3 (CST, 4947; 1:1000); TBK1 (CST, 3504T; 1:1000); p-TBK1 (CST, 5483; 1:1000); HSP90 (ABclonal, A5027; 1:5000); Elisa Kit: TGF-β (Solarbio, Cat: SEKM-0035, Beijing, China). Liver and salivary gland H&E-stained sections were independently scored by two pathologists blinded to group allocation. Hepatic injury was semi-quantitatively graded (0–3) for hepatocellular edema, perivascular inflammation, and fibrosis, and a composite score was calculated. Salivary gland lymphocytic infiltration was scored on a 0–4 focus score scale.

#### 2.1.4. Fluorescence Staining for ROS and Mitochondrial Membrane Potential

Frozen liver sections were prepared using a cryostat. For ROS detection, sections were washed with purified water, encircled with a hydrophobic barrier pen, and incubated with ROS staining solution (Beyotime, S0033, Shanghai, China) at 37 °C for 30 min in the dark. After washing with PBS, nuclei were counterstained with DAPI (Beyotime, B0025) for 10 min at room temperature. For JC-1 staining, sections were incubated with JC-1 working solution (prepared by diluting JC-1 stock in ultrapure water followed by JC-1 staining buffer according to the manufacturer’s instructions) at 37 °C for 20 min, then washed twice with JC-1 staining buffer. All sections were mounted with anti-fade mounting medium and imaged under a fluorescence microscope. ROS-positive cells were visualized using FITC settings. JC-1 aggregates were detected in the red channel, while JC-1 monomers were detected in the green channel.

#### 2.1.5. Inflammatory Cytokine Measurement

Concentrations of thirteen inflammatory cytokines in liver tissue were determined using the LEGENDplex™ Mouse Inflammation Panel (BioLegend, San Diego, CA, USA) according to the manufacturer’s instructions. This bead-based multiplex assay employs a sandwich immunoassay principle combined with flow cytometric detection. Briefly, samples were incubated with antibody-immobilized capture beads, followed by incubation with biotinylated detection antibodies and streptavidin-phycoerythrin (SA-PE).

#### 2.1.6. Transcriptomic and Metabolomic Profiling and 16S rDNA Gut Microbiota

Liver tissues from the Control, Model and High-dose trifolirhizin groups were snap-frozen for analysis. Total RNA was extracted using TRIzol, and libraries were sequenced on the BGI platform. Reads were aligned to mm10 with STAR; differential expression was determined by DESeq2 (|log_2_FC| > 1, adjusted *p* < 0.05). GSEA was performed against MSigDB gene sets. For metabolomics, tissue extracts were analyzed by UHPLC–Q Exactive HF-X MS in positive and negative modes. Data were processed with Compound Discoverer 3.2 and SIMCA. Differentially abundant metabolites were selected by VIP > 1, |log_2_FC| > 1, and *p* < 0.05. 16S rDNA Gut Microbiota experimental procedures are consistent with those in our previous work [13].

### 2.2. Cell Experiments

#### 2.2.1. CCK-8 Assay

The concentration and exposure time of H_2_O_2_ were selected based on previous reports [15,16] to establish an oxidative stress injury model in HepG2 cells. Cell viability was evaluated using the Cell Counting Kit-8 (CCK-8, Solarbio, CA1210, Beijing, China) according to the manufacturer’s instructions. HepG2 cells were seeded in 96-well plates at a density of 5 × 10^3^ cells per well and cultured overnight to allow attachment. Cells were then treated with varying concentrations of trifolirhizin (0, 5, 10, 15, 20, 40, 60 µM) for 8 h. After treatment, 10 µL of CCK-8 solution was added to each well, and the plates were incubated at 37 °C for 2 h. Absorbance was measured at 450 nm using a Multiskan FC microplate reader(SkanIt 7.1). Cell viability was calculated as the percentage of absorbance relative to the untreated control group.

#### 2.2.2. Cell Grouping

To determine the appropriate concentration range of trifolirhizin for subsequent experiments, cell viability was first assessed using the CCK-8 assay. The results showed that compared with the 5 µM group, the 10 µM group exhibited a significant increase in cell viability, while no significant differences were observed among the 10 µM, 15 µM, and 20 µM groups. Additionally, a potential difference was noted between the 20 µM and 40 µM groups (Supplementary Materials Figure S1).

Based on these findings, trifolirhizin concentrations of 15 µM, 20 µM, and 25 µM were selected for the subsequent mechanistic studies to ensure both efficacy and safety within the linear range of the dose–response curve. Cells were treated accordingly, and the culture medium was replaced every two days.

HepG2 cells were seeded in 6-well plates and divided into six groups: a control group (untreated), a model group treated with 200 μM H_2_O_2_ alone for 8 h, and three trifolirhizin treatment groups exposed to 200 μM H_2_O_2_ combined with 15 µM, 20 µM, or 25 µM trifolirhizin for 8 h, respectively. Glutathione (GSH) was used at a concentration of 1 mM, as previously described [17]. cGAMP was used at a concentration of 20 µM, as previously described [18].

#### 2.2.3. Antioxidant Enzyme Activity

Cell pellets were lysed by ultrasonication in extraction buffer and centrifuged, and the resulting supernatants were collected. Cell culture supernatants were used directly without further processing. The activities of glutathione peroxidase (GSH-Px), superoxide dismutase (SOD), and catalase (CAT) were determined using commercial kits (Solarbio, BC6275, BC5165, and BC0205, respectively).

#### 2.2.4. Assessment of Intracellular ROS and Mitochondrial Membrane Potential (ΔΨm)

Intracellular ROS levels were measured using the DCFH-DA probe (Solarbio, CA1410). Cells (1–2 × 10^6^/mL) in serum-free medium were loaded with 1:3000 DCFH-DA at 37 °C for 20 min (mixing every 3–5 min), washed three times, and analyzed by flow cytometry 10.10.0 (FACS Calibur, BD Canto II; Ex/Em 488/525 nm).

Mitochondrial membrane potential was assessed with the JC-1 assay (Solarbio, M8650). JC-1 working solution was prepared by diluting 200× JC-1 in ultrapure water (50 μL per 8 mL) and adding 5× staining buffer (2 mL per 8 mL). Cells (1 × 10^6^) were incubated with an equal volume of working solution at 37 °C for 20 min, washed twice with 1× staining buffer, and analyzed by flow cytometry (JC-1 monomers: Ex/Em 490/530 nm; aggregates: 525/590 nm).

#### 2.2.5. Seahorse XF Assays

Cellular oxygen consumption rate (OCR) and extracellular acidification rate (ECAR) were measured with a Seahorse XFe96 analyzer using Mito Stress Test (103015-100) and Glycolysis Stress Test (103020-100) kits. Sensor cartridge preparation: One day before assay, the cartridge was hydrated with sterile water at 37 °C in a non-CO_2_ incubator. On assay day, replace water with 200 µL/well XF Calibrant and incubate for 45–60 min at 37 °C (no CO_2_). Cell preparation: For the Mito Stress Test, cells were cultured in DMEM supplemented with glucose, pyruvate, and glutamine (all 100×). For the Glycolysis Stress Test, DMEM contained pyruvate and glutamine (100× each). Remove culture medium, leaving 20 µL, wash twice with 200 µL assay medium, then add 160 µL assay medium and incubate for 60 min at 37 °C (no CO_2_). Injections and measurements: Mito Stress Test: sequentially inject oligomycin (1.5 µM), FCCP (1.5 µM), and rotenone/antimycin A (0.5 µM). Glycolysis Stress Test: sequentially inject glucose (10 mM), oligomycin (2 µM), and 2-DG (50 mM). OCR and ECAR were recorded following the manufacturer’s instructions.

### 2.3. Statistical Analysis

All data are presented as mean ± SD. Normality and homogeneity of variance were assessed using Shapiro–Wilk and Levene’s tests, respectively. For multiple group comparisons, one-way ANOVA with Tukey’s post hoc test was applied for normally distributed data with equal variances; otherwise, Welch’s ANOVA (Games–Howell) or the Kruskal–Wallis test (Dunn’s post hoc) was used. Longitudinal data (blood glucose, body weight) were analyzed by two-way repeated measures ANOVA. By default, group comparisons were performed between the normal and model groups and between the model group and the low-, medium-, and high-dose groups; non-significant (ns) differences are not indicated. All statistical analyses were performed with GraphPad Prism 10.4. A two-tailed *p* < 0.05 was considered statistically significant.

## 3. Results

### 3.1. Trifolirhizin Attenuates Hepatic Inflammation

Compared with C57BL/6J controls, NOD mice exhibited significantly reduced stimulated salivary flow rates and increased daily water consumption, consistent with the classic pSS-like phenotype. Notably, high-dose trifolirhizin treatment (20 mg/kg/d) significantly improved salivary secretion and reduced polydipsia compared to the untreated NOD model group. These data indicate that trifolirhizin effectively attenuates the exocrine dysfunction characteristic of pSS in this model.

**Figure 1:**
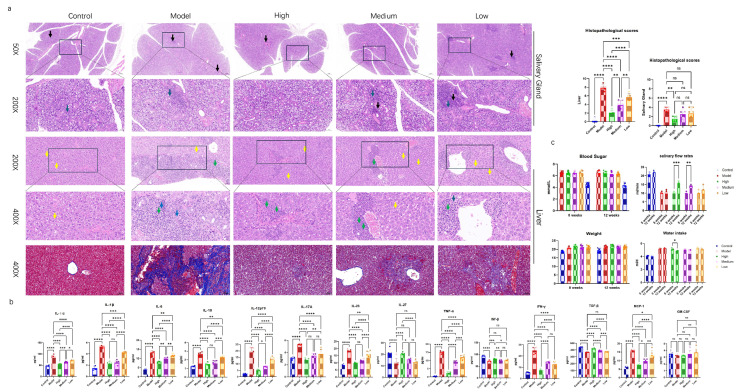
Trifolirhizin ameliorates hepatic and salivary gland pathological injury and regulates inflammatory cytokine profiles in NOD mice. (**a**) Representative H&E-stained liver and salivary gland and Histopathological Scores. Salivary Gland: Scale bars = 50 μm and 200 μm; Liver: Scale bars = 20 μm and 50 μm. (**b**) Quantitative analysis of multiple inflammatory cytokines. Data are presented as mean ± SD. (**c**) Body weight, blood glucose, salivary flow rate, and water intake before and after treatment (8 and 12 weeks). Data are presented as mean ± SD. * *p* < 0.05, ** *p* < 0.01, *** *p* < 0.001, **** *p* < 0.0001, ns *p* > 0.05.

H&E staining revealed that NOD mice (model group) exhibited marked hepatocellular edema (yellow arrows), extensive perivascular inflammatory infiltration (green arrows), and marked connective tissue proliferation (blue arrows), indicating severe hepatic pathology. In the high-dose group, hepatocellular edema was markedly improved (yellow arrows), with only mildly loosened cytoplasm and a small amount of peri-vascular inflammatory infiltration (green arrows). The medium-dose group showed slight improvement, displaying hepatocellular edema (yellow arrows) with mildly loosened cytoplasm, and a moderate amount of perivascular inflammatory infiltration (green arrows). In the low-dose group, hepatocellular edema persisted (yellow arrows), accompanied by abundant perivascular inflammatory infiltration (green arrows) and a small amount of connective tissue proliferation (blue arrows). Masson’s trichrome staining further demonstrated that in the model group, extensive hepatocellular necrosis and lysis were present, with nuclear pyknosis or karyorrhexis, residual cell debris, and infiltration of inflammatory cells and erythrocytes, replaced by large areas of blue-green collagen fiber deposition; fiber bundles extended from the portal area into the hepatic parenchyma, with bridging fibrosis in some areas, and the hepatic lobular architecture was disorganized. In the high-dose group, only focal hepatocellular necrosis and lysis were observed, along with nuclear pyknosis or karyorrhexis, residual cell debris, and inflammatory cell and erythrocyte infiltration, which were replaced by small areas of blue-green collagen fibers; the hepatic lobular architecture remained disorganized. The medium-dose group showed focal hepatocellular necrosis and lysis with similar features, replaced by medium-sized areas of blue-green collagen deposition, with fiber bundles extending from the portal area into the parenchyma and disorganized hepatic lobular structure. In the low-dose group, multifocal hepatocellular necrosis and lysis were evident, replaced by large areas of blue-green collagen fibers, with fiber bundles extending from the portal area and disorganized lobular architecture. Trifolirhizin treatment dose-dependently ameliorated these liver histological abnormalities, with the high-dose group showing only mild lesions (Figure 1a). In the salivary glands, H&E staining of the model group showed multifocal inflammatory cell infiltration (black arrows), with occasional atrophy and reduced volume of surrounding acinar cells. The high-dose trifolirhizin group displayed marked improvement, exhibiting only rare, small focal inflammatory cell infiltration (black arrows) and no obvious abnormalities in acinar cells. The medium-dose group also demonstrated notable improvement, characterized by rare, small focal inflammatory cell infiltration (black arrows) and occasional acinar cell atrophy (blue arrows) with reduced volume. Cytokine analysis using a cytometric bead array showed that, except for IL-17A and GM-CSF, levels of the measured inflammatory factors were significantly altered among groups. The high-dose trifolirhizin group exhibited the most pronounced regulatory effects, with significant alterations in all measured factors compared with the model group, and also differed significantly from the other treatment groups except for IL-10 and IL-1β (Figure 1b). These results collectively demonstrate that trifolirhizin effectively alleviates hepatic inflammation and fibrosis and ameliorates salivary gland pathology.

### 3.2. Trifolirhizin Preserves Mitochondrial Function and Inhibits ROS Accumulation in the Liver of NOD Mice

**Figure 2:**
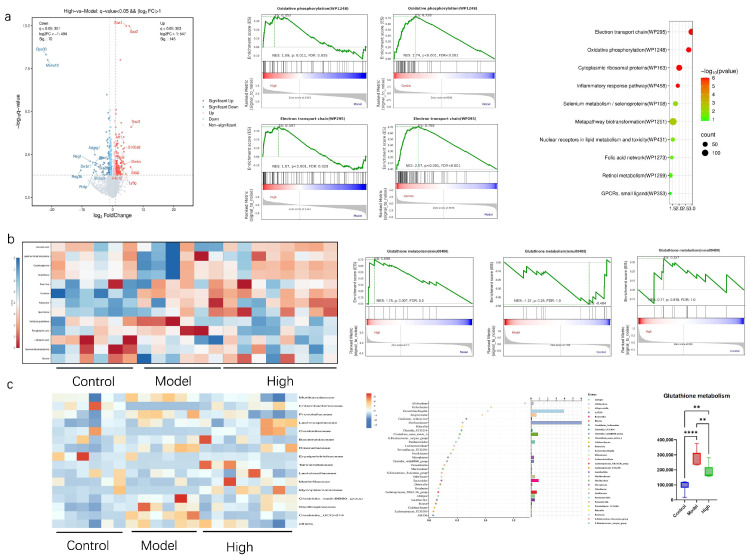
Multi-omics profiling of liver tissues from the NOD model and high-dose trifolirhizin groups. (**a**) (**Left**) Volcano plot of differentially expressed genes between the Model group and the High-dose group; (**Middle**) Transcriptomic GSEA revealing significant enrichment of the oxidative stress response gene set and the mitochondrial electron transport chain gene set in the Model vs. High-dose and Model vs. Control comparison. NES, normalized enrichment score; FDR, false discovery rate; (**Right**) Top 10 GESA-predicted pathways. (**b**) Metabolomic analysis. (**Left**): heatmap of the top 50 differentially abundant metabolites between the two groups. (**Right**): KEGG pathway enrichment analysis of the differential metabolites; (**c**) Gut microbiota alterations in NOD mice after high-dose trifolirhizin treatment. (**Left**) Top 15 differentially abundant bacterial orders (L5 level) between the Control, Model and High-dose groups. (**Middle**) Random forest analysis between the Control, Model and High-dose groups; (**Right**) PICRUSt2-predicted Glutathione metabolism pathways’ Difference between Control, Model and High-dose groups. ** *p* < 0.01, **** *p* < 0.0001.

Mitochondrial dysfunction and excessive ROS accumulation are core upstream drivers of autoimmune liver injury, which not only directly cause hepatocyte damage but also activate the downstream cGAS-STING inflammatory cascade. To gain insight into the hepatoprotective action of trifolirhizin, we first performed transcriptomic analysis of liver tissues from the Model and high-dose trifolirhizin groups. Independent GSEA of the transcriptomic data revealed that oxidative phosphorylation and mitochondrial electron transport chain were the top differentially enriched biological processes between the Model and Control groups (Figure 2a). Notably, gene sets associated with oxidative phosphorylation and the electron transport chain were significantly downregulated in the Model group compared with healthy controls. High-dose trifolirhizin treatment restored the expression of these genes, returning them toward the Control level.

Glutathione metabolism emerged as a shared signal across host and microbial compartments, albeit with distinct regulatory patterns. Metabolomic profiling revealed that glutathione metabolism did not differ significantly between the Model and Control groups, yet was significantly altered following high-dose trifolirhizin treatment compared with the Model group (Figure 2b). Notably, the treated group also showed no significant difference from the Control group, indicating that the drug-induced shift was moderate and did not drive glutathione metabolism beyond the physiological range. In the gut microbiota, metagenomic functional prediction of fecal DNA revealed a more pronounced remodeling: glutathione metabolism was significantly altered both between the Model and Control groups and between the Model and High-dose groups (Figure 2c). Importantly, the microbial glutathione metabolism signature in the High-dose group remained significantly different from that of the Control group. These findings suggest that glutathione metabolism is differentially engaged across tissues—the liver maintains partial homeostatic control that is reinforced by trifolirhizin, whereas the gut microbiota undergoes more extensive remodeling that does not simply revert to the healthy baseline. The co-occurrence of glutathione pathway alterations in both compartments points to a coordinated, yet compartment-specific, redox metabolic response to trifolirhizin treatment in autoimmune liver injury. 

**Figure 3:**
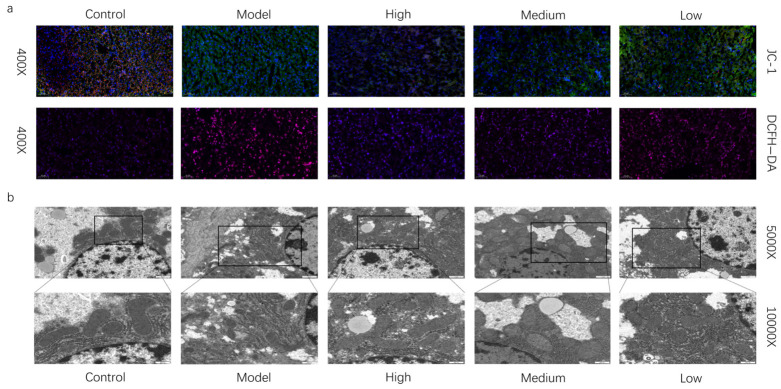
Effects of Trifolirhizin on mitochondrial function and ultrastructure. (**a**) Fluorescence staining results: JC-1 (**top row**) and DCFH-DA (**bottom row**) staining in Control, Model, and treatment groups (High, Medium, Low). (**b**) TEM images showing mitochondrial ultrastructure in each group. Scale bars = 500 nm and 1 μm.

These multi-omics findings prompted us to directly evaluate mitochondrial integrity and redox status in the liver. We therefore examined ΔΨm, ROS levels, and mitochondrial ultrastructure in the liver tissues of NOD mice. As shown in Figure 3a, JC-1 staining revealed that the Control group exhibited strong red/orange fluorescence, indicating intact mitochondrial function and high ΔΨm. In contrast, the Model group showed a dramatic shift to green fluorescence, confirming severe mitochondrial depolarization and ΔΨm collapse. Trifolirhizin treatment dose-dependently restored ΔΨm: the High-dose group displayed fluorescence intensity comparable to the Control group, while the Medium and Low-dose groups exhibited gradual recovery of red/orange fluorescence with residual green signal. For ROS detection, DCFH-DA staining demonstrated that the Model group had markedly enhanced fluorescence, reflecting massive ROS accumulation in hepatic tissue. Trifolirhizin treatment significantly reduced ROS fluorescence intensity in a dose-dependent manner, with the High-dose group showing the most potent ROS-scavenging effect, nearly returning to the Control level.

Transmission electron microscopy (TEM) was performed to directly visualize the ultrastructural consequences of these functional changes (Figure 3b). In the Control group, mitochondria presented normal morphology with intact double membranes and clear, dense cristae. The Model group exhibited severe mitochondrial damage, including marked swelling, vacuolization, membrane rupture, and cristae disappearance, consistent with the ΔΨm collapse and ROS overproduction described above. High-dose trifolirhizin treatment effectively reversed these ultrastructural injuries: mitochondria displayed intact membranes, well-organized cristae, and normal morphology, closely resembling the Control group. The Medium-dose group showed moderate structural improvement, while the Low-dose group still exhibited obvious mitochondrial swelling and structural damage, similar to the Model group.

Taken together, these multi-omics and imaging findings systematically establish that trifolirhizin restores hepatic redox homeostasis and mitochondrial bioenergetics. The transcriptional restoration of oxidative phosphorylation and electron transport chain genes, the coordinated alterations in glutathione metabolism across liver and gut microbiota, and the structural and functional preservation of mitochondria converge to identify redox regulation and mitochondrial integrity as the central targets of trifolirhizin in autoimmune liver injury.

### 3.3. Regulatory Effects of Trifolirhizin on the cGAS-STING Inflammatory Axis in the NOD Mouse Model

The cGAS-STING signaling axis is a central regulator of innate immune and inflammatory responses, whose aberrant activation is critically implicated in the pathogenesis of autoimmune diseases in NOD mice. To elucidate the immunomodulatory mechanism of Trifolirhizin in the NOD model, we evaluated the protein expression of core components in the cGAS-STING signaling pathway using Western blotting.

**Figure 4:**
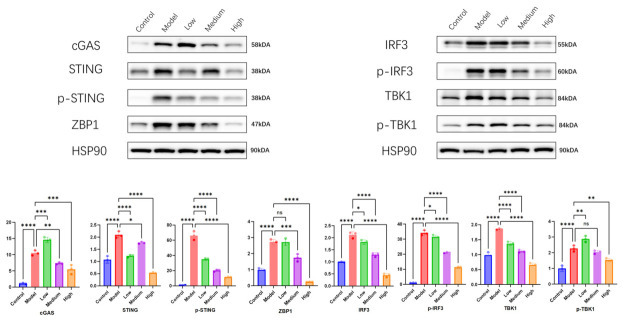
Effects of Trifolirhizin on the protein expression of key molecules in the cGAS-STING-TBK1-IRF3 signaling pathway in NOD mice detected by Western blotting. Representative Western blot bands of cGAS, STING, phosphorylated STING (p-STING), Z-DNA binding protein 1 (ZBP1), IRF3, phosphorylated IRF3 (p-IRF3), TBK1, and phosphorylated TBK1 (p-TBK1), with HSP90 serving as the loading control. Data are presented as mean ± SD. * *p* < 0.05, ** *p* < 0.01, *** *p* < 0.001, **** *p* < 0.0001; ns, no significant difference.

As shown in Figure 4, compared with the normal Control group, the NOD model group exhibited a remarkable upregulation in the protein levels of cGAS, total STING, phosphorylated STING (p-STING), and ZBP1 (*p* < 0.0001), indicating robust activation of the cGAS-STING pathway in the NOD model. Treatment with Trifolirhizin exerted a dose-dependent regulatory effect on these proteins: the Low-dose Trifolirhizin group showed no inhibitory effect, and even further increased cGAS expression compared with the Model group; in contrast, Medium and High doses of Trifolirhizin significantly downregulated the protein levels of cGAS, p-STING, and ZBP1 (*p* < 0.01, *p* < 0.001 or *p* < 0.0001 vs. Model group).

We further examined the downstream effector molecules of the cGAS-STING pathway, namely TBK1 and IRF3. Compared with the Control group, the protein levels of total IRF3, phosphorylated IRF3 (p-IRF3), total TBK1, and phosphorylated TBK1 (p-TBK1) were all significantly elevated in the Model group (*p* < 0.05, *p* < 0.01 or *p* < 0.0001), confirming the excessive activation of the downstream inflammatory cascade in NOD mice. Consistent with the upstream molecules, Low-dose Trifolirhizin did not suppress the upregulation of these proteins, and even slightly increased the expression of p-IRF3, total TBK1, and p-TBK1 compared with the Model group. In contrast, Medium and High doses of Trifolirhizin significantly inhibited the phosphorylation of TBK1 and IRF3 in a dose-dependent manner, with marked reductions in p-TBK1 and p-IRF3 levels observed in the High-dose groups (*p* < 0.01, *p* < 0.001 or *p* < 0.0001 vs. Model group). It is worth noting that there was no significant difference in p-TBK1 expression between the Medium-dose Trifolirhizin group and the Model group, suggesting that Trifolirhizin mainly modulates TBK1 activity by inhibiting its phosphorylation, rather than affecting its total protein expression.

Taken together, these findings demonstrate that Medium and High doses of Trifolirhizin can effectively inhibit the hyperactivation of the cGAS-STING inflammatory signaling pathway in NOD mice, which may serve as a key molecular mechanism underlying the protective effect of Trifolirhizin against autoimmune injury in the NOD model.

### 3.4. High-Dose Trifolirhizin (25 µM) Optimally Protects Antioxidant Enzymes and Suppresses the cGAS-STING Axis in H_2_O_2_-Treated HepG2 Cells

Oxidative stress is driven by the imbalance between reactive oxygen species accumulation and cellular antioxidant defense capacity, where SOD, CAT, and GSH-Px are the core endogenous antioxidant enzymes that maintain cellular redox homeostasis [17]. To evaluate the antioxidant effect of Trifolirhizin and screen the optimal intervention dose for subsequent mechanistic studies, we first detected the activities of these three antioxidant enzymes in H_2_O_2_-induced oxidative stress HepG2 cells treated with gradient doses of Trifolirhizin.

**Figure 5:**
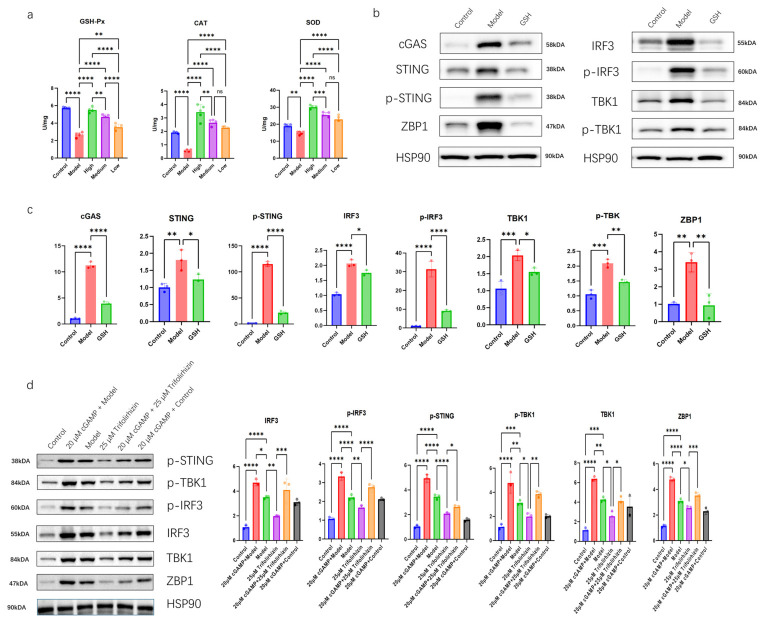
Trifolirhizin enhances cellular antioxidant enzyme activity in a dose-dependent manner and inhibits hyperactivation of the cGAS-STING signaling pathway in H_2_O_2_-induced oxidative stress HepG2 cells, with high-dose Trifolirhizin showing the optimal antioxidant efficacy. (**a**) Quantitative analysis of the activities of core antioxidant enzymes: superoxide dismutase (SOD), catalase (CAT), and glutathione peroxidase (GSH-Px) in HepG2 cells. (**b**) Representative Western blot bands of cGAS, STING, phosphorylated STING (p-STING), ZBP1, IRF3, phosphorylated IRF3 (p-IRF3), TBK1, and phosphorylated TBK1 (p-TBK1) in Control, Model, and GSH groups, with HSP90 serving as the loading control. (**c**) Quantitative analysis of the relative expression levels of target proteins, normalized to HSP90. Data are presented as mean ± SD. * *p* < 0.05, ** *p* < 0.01, *** *p* < 0.001, **** *p* < 0.0001; ns, no significant difference. (**d**) Pharmacological rescue experiment using the cGAS-specific agonist 2′,3′-cGAMP. Representative Western Blot images (**left**) and quantitative analysis (**right**) of cGAS-STING-ZBP1 pathway protein expression in HepG2 cells under different treatments. HSP90 was used as the loading control. Data are presented as mean ± SD (n = 3) * *p* < 0.05, ** *p* < 0.01, *** *p* < 0.001, **** *p* < 0.0001.

As shown in Figure 5a, compared with the normal Control group, the H_2_O_2_-stimulated Model group exhibited a dramatic and significant reduction in the activities of SOD, CAT, and GSH-Px, indicating severe impairment of the cellular antioxidant system under oxidative stress. Trifolirhizin treatment exerted a dose-dependent protective effect on antioxidant enzyme activities: Low and Medium doses of Trifolirhizin moderately but significantly increased the activities of SOD, CAT, and GSH-Px compared with the Model group, while High-dose (25 µM) Trifolirhizin showed the most potent recovery effect. Notably, the activities of SOD and CAT in the High-dose Trifolirhizin group were significantly better than those of the Medium and Low-dose groups. Given the optimal antioxidant performance of High-dose Trifolirhizin in restoring cellular antioxidant defense capacity, we selected the high-dose intervention for subsequent molecular mechanism studies.

To determine whether ROS serves as a key upstream trigger for the cGAS-STING pathway, we examined the effect of glutathione (GSH) in H_2_O_2_-challenged HepG2 cells, a well-established model of oxidative stress. As shown in Figure 5b,c, the protein levels of cGAS, total STING, p-STING, and ZBP1 were markedly elevated in the Model group compared with the Control group, confirming that H_2_O_2_-derived ROS are sufficient to activate this pathway. This activation was functionally propagated to downstream signaling, evidenced by significantly increased phosphorylation of TBK1 and IRF3 in the Model group. Moreover, GSH similarly attenuated cGAS-STING activation, further reinforcing that the removal of ROS is necessary and sufficient to block this pathway (Figure 5b,c).

We next sought to determine whether Trifolirhizin exerts its protective effect specifically through targeting this pathway, using a pharmacological rescue experiment with the selective cGAS agonist 2′,3′-cGAMP (hereinafter referred to as cGAMP).

As shown in Figure 5d, consistent with our previous results, H_2_O_2_ stimulation significantly upregulated the protein expression of total IRF3, TBK1, and ZBP1, as well as the phosphorylation levels of STING (p-STING), TBK1 (p-TBK1), and IRF3 (p-IRF3) in the Model group compared with the Control group (all **** *p* < 0.0001). Treatment with 20 μM cGAMP further exacerbated the activation of the cGAS-STING pathway in H_2_O_2_-challenged cells, leading to an even more pronounced increase in the expression of all detected proteins compared with the Model group (** *p* < 0.01 or *** *p* < 0.001). In contrast, 25 μM Trifolirhizin treatment significantly attenuated the abnormal activation of the cGAS-STING-ZBP1 pathway induced by H_2_O_2_, reducing the expression levels of these proteins to near-normal levels.

Most critically, the rescue experiment revealed that co-treatment with cGAMP completely reversed the inhibitory effect of Trifolirhizin on the cGAS-STING pathway. The protein expression levels of p-STING, p-TBK1, p-IRF3, total IRF3, TBK1, and ZBP1 in the cGAMP + Trifolirhizin co-treatment group were significantly higher than those in the Trifolirhizin alone treatment group (** *p* < 0.01 or *** *p* < 0.001) and were statistically indistinguishable from those in the cGAMP alone treatment group. Additionally, treatment of normal HepG2 cells with cGAMP alone only slightly activated the cGAS-STING pathway, with a much lower activation degree than that observed in the H_2_O_2_-induced Model group.

These results provide pharmacological evidence that trifolirhizin acts upstream of STING, most likely by preserving mitochondrial integrity and limiting ROS/mtDNA leakage, thereby suppressing cGAS-STING signaling. The ability of cGAMP to abrogate the protective effect of trifolirhizin confirms that the cGAS-STING pathway is functionally required for its action, downstream of mitochondrial protection.

### 3.5. Trifolirhizin Mitigates Oxidative Stress Damage in HepG2 Cells Through Modulating Mitochondrial Function and Intracellular ROS Levels

**Figure 6:**
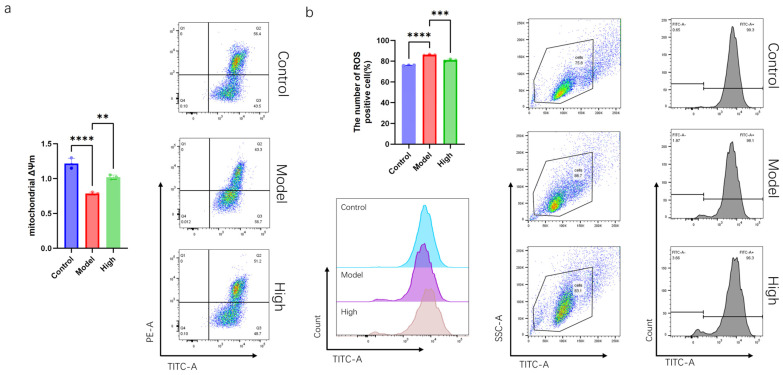
Effects of Trifolirhizin on ΔΨm and ROS levels in H_2_O_2_-induced oxidative stress HepG2 cell models detected by flow cytometry. (**a**) ΔΨm analysis by JC-1 staining. (**Left**): Quantitative analysis of ΔΨm levels in Control, Model (oxidative stress-induced), and High-dose Trifolirhizin-treated HepG2 cells. (**Right**): Representative flow cytometry dot plots for each group. (**b**) Intracellular ROS levels detected by DCFH-DA staining. (**Upper**): Quantitative analysis of ROS-positive cell percentages. (**Lower left**): Overlay histogram of ROS fluorescence intensity across groups. (**Middle**): Representative scatter plots for cell gating. (**Right**): Histograms of FITC fluorescence intensity for ROS-positive cells. Data are presented as mean ± SD. ** *p* < 0.01, *** *p* < 0.001, **** *p* < 0.0001 vs. Model group.

To investigate the protective mechanism of Trifolirhizin against oxidative stress in hepatocytes, flow cytometry was performed to assess mitochondrial function and intracellular ROS levels in H_2_O_2_-induced oxidative stress HepG2 cell models. As shown in Figure 6a, JC-1 staining was used to evaluate ΔΨm, a critical indicator of mitochondrial integrity and function. Compared with the untreated Control group, the Model group exhibited a significant reduction in ΔΨm, confirming severe mitochondrial damage triggered by oxidative stress in HepG2 cells. Notably, treatment with high-dose Trifolirhizin effectively restored ΔΨm levels, with a statistically significant increase compared to the Model group, demonstrating its protective effect on mitochondrial function in hepatocytes.

For intracellular ROS detection, DCFH-DA staining was applied to quantify ROS-positive cells. As presented in Figure 6b, the Model group showed a marked elevation in the percentage of ROS-positive cells, verifying the successful establishment of the oxidative stress model in HepG2 cells. Trifolirhizin treatment significantly decreased the proportion of ROS-positive cells, accompanied by a leftward shift in the ROS fluorescence intensity histogram, which further confirmed its robust ROS-scavenging activity in hepatocytes. Collectively, these results indicate that Trifolirhizin alleviates oxidative stress injury in HepG2 cells by inhibiting intracellular ROS accumulation and preserving mitochondrial membrane potential, highlighting its potential as a hepatoprotective agent against oxidative stress-related liver damage.

### 3.6. Trifolirhizin Restores Glycolytic Function and Mitochondrial Respiratory Capacity in Oxidative Stress-Injured HepG2 Cells

Cellular energy homeostasis, mainly sustained by glycolysis and mitochondrial oxidative phosphorylation (OXPHOS), is tightly coupled with cellular redox state, and mitochondrial dysfunction is a core hallmark of oxidative stress-induced hepatocyte injury [18]. To further elucidate the protective mechanism of Trifolirhizin against oxidative stress in HepG2 cells, we performed a Seahorse extracellular flux assay to systematically evaluate the effects of Trifolirhizin on glycolytic function and mitochondrial respiratory capacity.

**Figure 7:**
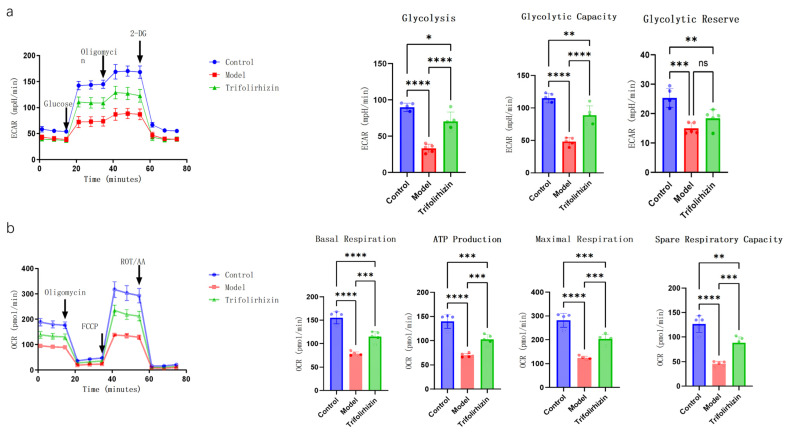
Trifolirhizin restores glycolytic function and mitochondrial respiratory capacity in H_2_O_2_-induced oxidative stress-injured HepG2 cells, as detected by Seahorse extracellular flux assay. (**a**) Extracellular acidification rate (ECAR) measurement and quantitative analysis of glycolysis-related parameters. (**Left**): Real-time ECAR dynamic curve with sequential injections of glucose, oligomycin, and 2-deoxy-D-glucose (2-DG). (**Right**): Quantitative analysis of core glycolytic indicators, including Glycolysis, Glycolytic Capacity, and Glycolytic Reserve. (**b**) Oxygen consumption rate (OCR) measurement and quantitative analysis of mitochondrial respiration-related parameters. (**Left**): Real-time OCR dynamic curve with sequential injections of oligomycin, carbonyl cyanide 4-(trifluoromethoxy)phenylhydrazone (FCCP), and rotenone/antimycin A (ROT/AA). (**Right**): Quantitative analysis of core mitochondrial respiratory indicators, including Basal Respiration, ATP Production, Maximal Respiration, and Spare Respiratory Capacity. Data are presented as mean ± SD. * *p* < 0.05, ** *p* < 0.01, *** *p* < 0.001, **** *p* < 0.0001; ns, no significant difference.

As shown in Figure 7a, extracellular acidification rate (ECAR) was continuously monitored to assess glycolytic function via sequential drug intervention. Compared with the normal Control group, the H_2_O_2_-induced Model group exhibited a dramatic and global impairment of glycolytic function, with significant reductions in Glycolysis, Glycolytic Capacity, and Glycolytic Reserve. These results indicated that oxidative stress severely disrupted the glycolytic energy supply system in HepG2 cells. Encouragingly, High-dose Trifolirhizin treatment effectively reversed these glycolytic defects: it markedly elevated the levels of Glycolysis and Glycolytic Capacity compared with the Model group, demonstrating that Trifolirhizin effectively preserved the glycolytic function and adaptive capacity of hepatocytes under oxidative stress.

We further evaluated mitochondrial oxidative phosphorylation function by measuring the OCR (Figure 7b). Compared with the Control group, the Model group showed severe mitochondrial respiratory dysfunction, with remarkable decreases in all core respiratory indicators: Basal Respiration, ATP Production, Maximal Respiration, and Spare Respiratory Capacity. These findings confirmed that oxidative stress caused irreversible damage to mitochondrial electron transport chain function, leading to defective mitochondrial energy production and reduced stress tolerance in HepG2 cells. Notably, Trifolirhizin treatment significantly mitigated this mitochondrial injury: it markedly upregulated all the above mitochondrial respiratory parameters compared with the Model group, indicating that Trifolirhizin could effectively restore mitochondrial oxidative phosphorylation capacity, maintain cellular ATP supply, and enhance the respiratory reserve capacity of hepatocytes under oxidative stress.

Collectively, these results demonstrate that Trifolirhizin can preserve both glycolytic function and mitochondrial respiratory capacity in oxidative stress-injured HepG2 cells, thereby maintaining cellular energy homeostasis, which is highly consistent with our previous findings that Trifolirhizin protects mitochondrial membrane potential and inhibits intracellular ROS accumulation.

## 4. Discussion

In the present study, we systematically demonstrated that trifolirhizin not only ameliorates pSS-like manifestations—as evidenced by improved salivary flow and reduced polydipsia—but also attenuates the associated hepatic injury in NOD mice. The hepatoprotective effect is underpinned by preservation of mitochondrial integrity and suppression of the ROS/cGAS-STING signaling cascade.

The NOD mouse is a well-established spontaneous model that recapitulates both primary Sjögren’s syndrome (pSS)-like exocrinopathy and systemic autoimmune features. In female NOD mice, overt type 1 diabetes (T1D) typically develops after 12–14 weeks of age [19]. Notably, early hepatic injury in NOD mice has been shown to be accompanied by oxidative stress and mitochondrial damage [20,21]. In the present study, we therefore selected an 8–12-week treatment window, a period that precedes the onset of overt diabetes in this strain. Blood glucose monitoring confirmed that all NOD groups remained normoglycemic throughout the experiment, thereby excluding confounding metabolic improvement. This design allows us to specifically investigate autoimmune-driven, rather than hyperglycemia-driven, liver injury in the context of pSS.

A central finding of this study is the remarkable preservation of mitochondrial structure and function by trifolirhizin in the face of autoimmune oxidative stress. Mitochondrial swelling, cristae loss, and membrane rupture, as visualized by TEM, are hallmarks of severe hepatocyte injury in the NOD model. Trifolirhizin reversed these ultrastructural defects, restored ΔΨm, and effectively scavenged excessive ROS in both liver tissue and cultured hepatocytes. Crucially, we extended these morphological and biochemical observations to the functional level: Seahorse extracellular flux analysis demonstrated that trifolirhizin rescues both glycolytic function and mitochondrial respiratory capacity in oxidatively stressed HepG2 cells. The significant recovery of basal respiration, ATP production, maximal respiration, and spare respiratory capacity indicates that trifolirhizin not only protects the structural integrity of mitochondria but also preserves the bioenergetic flexibility required for hepatocyte survival under stress. These functional data provide a robust mechanistic bridge between the observed ultrastructural improvements and the attenuation of cell death and inflammation.

To gain a systems-level understanding of the protective mechanism, we performed integrated transcriptomic and metabolomic profiling of liver tissues [22]. The multi-omics data independently converged on pathways central to our hypothesis: gene set enrichment analysis highlighted oxidative stress responses and the mitochondrial electron transport chain as the most significantly altered biological processes after trifolirhizin treatment, while metabolomic analysis pinpointed glutathione metabolism as the core differentially regulated pathway. ROS are widely regarded as detrimental factors involved in cellular dysfunction [23,24]. The restoration of glutathione levels and the transcriptional upregulation of oxidative phosphorylation genes provide convergent evidence that trifolirhizin rewires the hepatic redox and energy homeostasis networks disrupted by autoimmune inflammation. This approach not only corroborates our targeted measurements of antioxidant enzyme activities (SOD, CAT, GSH-Px) but also reveals the broader metabolic landscape upon which trifolirhizin exerts its protective effects. The independent enrichment of oxidative phosphorylation in the gut microbiota provides additional systems-level support for a redox-centric mechanism. Although prokaryotic oxidative phosphorylation differs structurally from mitochondrial OXPHOS, its prominence as the top-ranked altered microbial pathway implies a broad “energy-redox remodeling” effect of trifolirhizin. Given the gut–liver metabolic axis, it is plausible that restoration of microbial energy metabolism contributes to the systemic antioxidant environment and the replenishment of GSH precursors, thereby indirectly reinforcing hepatic mitochondrial protection.

The mechanistic link between mitochondrial protection and suppression of innate immune signaling was established through our investigation of the cGAS-STING pathway. We found that the cGAS-STING axis was robustly activated in the livers of NOD mice, as evidenced by upregulated cGAS, phosphorylated STING, ZBP1, and the downstream phosphorylation of TBK1 and IRF3. Trifolirhizin dose-dependently suppressed this hyperactivation. In vitro, H_2_O_2_-derived ROS were sufficient to trigger the cGAS-STING pathway, and the antioxidant glutathione similarly attenuated this activation, confirming that ROS is a requisite upstream signal. The pharmacological rescue experiment using the cGAS agonist cGAMP further demonstrated that exogenous cGAMP overrides the inhibitory effect of trifolirhizin on STING, TBK1, and IRF3 phosphorylation, indicating that trifolirhizin acts at or upstream of cGAS to prevent signal propagation along this axis. However, we explicitly note that while the cGAMP rescue experiment establishes the functional relevance of the cGAS-STING pathway to trifolirhizin’s protective effects, it does not prove that this axis is the exclusive or indispensable mediator. The restoration of pathway activity by exogenous cGAMP could equally be explained by the fact that cGAMP bypasses the mitochondrial protection afforded by trifolirhizin, rather than indicating direct pharmacological targeting of cGAS itself. It remains plausible that the primary action of trifolirhizin lies in preserving mitochondrial membrane integrity and reducing mtDNA/ROS leakage, with the consequent dampening of cGAS-STING signaling being a critical downstream consequence. Definitive discrimination between these possibilities will require complementary loss-of-function approaches, such as siRNA-mediated silencing or genetic knockout of cGAS or STING, which constitute the logical next step of this investigation.

At the lowest dose of trifolirhizin, we observed an unexpected pattern: cGAS protein expression was increased, whereas STING expression was decreased; moreover, p-IRF3, total TBK1, and p-TBK1 were not reduced and even mildly elevated compared with the model group. Importantly, this paradoxical upregulation was not observed in the rescue experiments, suggesting that it is unlikely to reflect a canonical pharmacological action of trifolirhizin on the cGAS–STING axis. We therefore speculate that this low-dose phenomenon may be attributable to two interrelated factors. First, low-dose trifolirhizin may induce mild cellular stress or subclinical toxicity, which could non-specifically perturb intracellular homeostasis and trigger compensatory innate immune responses, including cGAS upregulation. Second, natural small molecules frequently exhibit polypharmacology, and dose-dependent multi-target effects are also commonly observed, as exemplified by metformin’s activation of AMPK [25,26]. At low concentrations, trifolirhizin might engage off-target pathways that intersect with TBK1–IRF3 signaling independently of STING, while its primary on-target STING inhibition becomes dominant only at higher doses. Consistent with this interpretation, the discrepancies disappeared at medium and high doses and were absent in the rescue setting. Taken together, the low-dose effects likely represent off-target or stress-related responses rather than a specific regulatory feature of trifolirhizin on the cGAS–STING pathway. Caution is therefore warranted when interpreting pharmacological effects observed only at the lowest dose.

The present study has several additional limitations that should inform future work. First, although we focused on the liver as a mitochondria-rich model organ well-suited for mechanistic validation, we did not evaluate the protective efficacy of trifolirhizin in the canonical target organs of NOD mice, specifically the pancreas and salivary glands. Establishing whether the mitochondrial protection observed in hepatocytes translates to these tissues will be essential for determining the full therapeutic potential of trifolirhizin in pSS and T1DM. Second, pharmacokinetic and long-term toxicity studies of trifolirhizin were not conducted, and its in vivo metabolic fate and chronic safety profile remain to be clarified before clinical translation can be contemplated. Third, beyond the cGAS-STING axis, other ROS-responsive innate immune sensors (e.g., NLRP3 inflammasome) may also be modulated by trifolirhizin and contribute to its anti-inflammatory effects; broader pathway profiling is warranted. Another limitation of the present study is that we did not include a drug withdrawal phase; therefore, the durability of the beneficial effects of trifolirhizin on liver integrity and function after treatment cessation remains unknown. Future studies incorporating a washout or post-treatment follow-up design will be necessary to determine the persistence of its hepatoprotective action. Furthermore, while we monitored blood glucose and body weight without detecting inter-group differences, more comprehensive metabolic profiling (e.g., fructosamine, insulin levels) would further strengthen the conclusion that the hepatoprotective effects are independent of glycemic control. Future studies should also examine the effect of trifolirhizin on other pSS-target organs, such as the lacrimal glands and pancreas, to comprehensively assess its therapeutic potential.

## 5. Conclusions

In conclusion, Trifolirhizin alleviates pSS-like manifestations and the associated liver injury by preserving mitochondrial integrity and suppressing the ROS/cGAS-STING inflammatory cascade. These findings position trifolirhizin as a promising natural candidate for treating pSS and its hepatic complications. Our findings provide a preclinical proof-of-concept for targeting mitochondrial health as a strategy to interrupt the vicious cycle of organ damage in systemic autoimmune diseases. Future investigations combining genetic manipulation of the cGAS-STING pathway, multi-organ efficacy assessment, and pharmacokinetic profiling will be critical to advance trifolirhizin toward clinical development for autoimmune complications.

## Data Availability

The raw sequence data reported in this study were deposited in the Genome Sequence Archive, National Genomics Data Center, China National Center for Bioinformation/Beijing Institute of Genomics, Chinese Academy of Sciences (GSA: CRA044596), and are publicly accessible at https://ngdc.cncb.ac.cn/gsa (accessed on 24 June 2026) [27].

## Supplementary Materials

The following supporting information can be downloaded at: https://www.mdpi.com/article/10.3390/antiox15070814/s1, Figure S1: Effect of Trifolirhizin concentration on cell survivalrate(CCK8).
