# Supplementary material for: Mitochondrial Protection by Trifolirhizin Alleviates Primary Sjögren’s Syndrome and Liver Injury via Coordinated Suppression of the ROS/cGAS-STING Pathway
Source: Antioxidants (Basel). 2026 Jun 28;15(7):814. doi: 10.3390/antiox15070814 (PMC13403522; doi:10.3390/antiox15070814)
Supplement: Supplementary file 1 [file antioxidants-15-00814-s001.zip › antioxidants-4345462-supplementary.pdf]

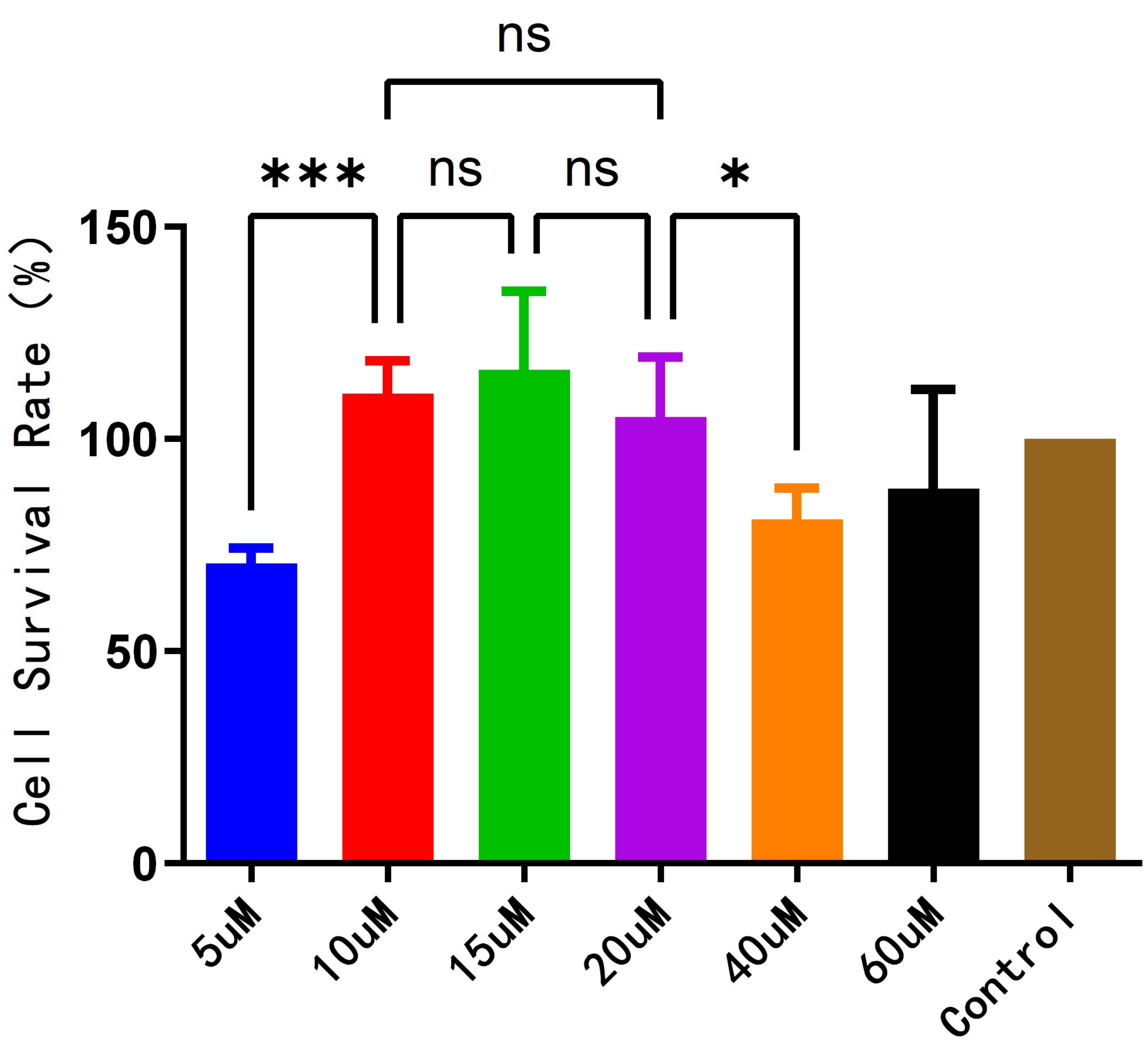

Figure S1. Effect of Trifolirhizin concentration on cell survival rate(CCK8).
Cell survival rates were evaluated in cells treated with various concentrations of the compound (5, 10, 15, 20, 40, and 60 μM) compared to an untreated control. Data are expressed as the mean ± standard deviation (SD). Statistical significance was analyzed using one-way ANOVA with post-hoc tests. Asterisks indicate statistically significant differences between the connected groups * $p < 0.05$, *** $p < 0.001$, ns, no significant difference.
